# The Ugandan Youth Quality of Life index: assessing the relevance of incorporating perceived importance into the quality of life measure and factors associated with the quality of life among youth in slum areas of Kampala, Uganda

**DOI:** 10.3402/gha.v9.31362

**Published:** 2016-07-15

**Authors:** Andre M. N. Renzaho, Joseph Kihika Kamara, Gilbert Kamanga

**Affiliations:** 1 School of Social Sciences and Psychology, Western Sydney University, Sydney, Australia; 2 Department of Epidemiology and Preventive Medicine, Monash University, Melbourne, Australia; 3 Humanitarian and Emergency Affairs – Southern Africa Region, World Vision International, Johannesburg, South Africa; 4 World Vision Uganda, Kampala, Uganda

**Keywords:** quality of life, Uganda, youth, international aid and development, resource-poor settings

## Abstract

**Background:**

While quality of life (QoL) has long been an explicit policy goal for international development programmes, no instruments have specifically been developed for measuring health-related QoL in resource-limited settings. The aim of this study was to develop and validate a QoL instrument for use in international aid and development programmes and to assess factors associated with QoL among youth participating in a civic engagement project in Kampala.

**Design:**

Using systematic random sampling, data were collected on 663 participants aged between 13 and 24 years in Kampala. The QoL questionnaire included 36 questions divided into a two-part scale: 18 questions rated for satisfaction (Part 1) and 18 other questions rated on importance (Part 2). The total sample was randomly divided into two split-half samples: one for the exploratory factor analysis (EFA; *N*=310) and the other for the confirmatorty factor analysis (CFA; *N*=353). The effect of demographic, socio-economic, and lifestyle factors on QoL was assessed using linear regressions.

**Results:**

The EFA yielded three factors: living conditions and lifestyle (seven items, *α*=0.84), social relationships (five items, *α*=0.86), and personal independence (five items, *α*=0.76). In the CFA, the initial model demonstrated a poor to marginal fit model. Its re-specification by examining modification indices resulted in a good model fit: Comparative Fit Index=0.95, Root Mean Square Error of Approximation=0.06, and p of Close Fit >0.05. The model incorporating perceived importance had lower Akaike Information Criteria and Bayesian Information Criteria values than the unweighted model, thereby providing very strong support to weight satisfaction scores with importance ratings when measuring QoL in Uganda. Poor QoL was associated with poor educational attainment, drug and substance misuse, and family disruption.

**Conclusions:**

The findings suggest that there is a relationship between QoL and lifestyle and structural issues among youth in Uganda. The study provides the first validated QoL measure to allow government and non-government organisations in low- and middle-income countries to track progress of international aid and development programmes.

## Introduction

Although the concept of ‘quality of life’ (QoL) was a subject of discussion in Plato and Aristotle's works (1, 2), research on QoL did not dominate the literature as a separate area of research until the 1960s. Since then, research on QoL has grown exponentially and the concept has been embraced in various research fields including sociology, economics, psychology, political science but predominantly in the field of health care and service delivery in industrialised countries (3). However, there have been controversies related to scales used in QoL measures, with the debate centring around whether satisfaction with life scores should be weighted for importance rating/ranking. Various research studies have proposed that it is undesirable and unnecessary to weight satisfaction scores by importance ratings (4, 5). Using different internationally known weighting algorithms, various researchers have found that importance ratings did not moderate the relationship between satisfaction scores and the overall life satisfaction (5–7). These studies theorise that satisfaction ratings already take into account the importance of the item, and therefore weighting satisfaction scores by importance rating/ranking is redundant (5).

In contrast, some authors stipulate that individuals perceive some aspects of their life to be more important, or to carry more weight, than others, and hence aspects of life are not of equal importance to all respondents in a survey and in any settings (8–10). They argue that by summing or averaging satisfaction scores across all QoL items, one implicitly assumes equal weight among items which is somewhat counterintuitive (10). In a series of studies, Hsieh (10–12) has consistently shown the merit and the need to weight satisfaction scores by importance rating/ranking.

However, most of the above studies have predominantly been carried out in industrialised countries without taking into account the cultural relativity of the QoL concept (13, 14). In industrialised countries where autonomy and independence are the main features, a high QoL equates to individual success, achievement, self-actualisation, and self-respect (13). In contrast, collectivist cultures found in low- and middle-income countries (LMICs) value interdependence, fulfilling roles within a group individuals belong to, and loyalty (14). In these cultures, a high QoL is constructed much more in family and group terms (13). Therefore, the assumptions that govern QoL and the importance attached to specific life events in one culture may not hold in another, thus highlighting the need to further test whether weighting satisfaction scores by importance rating is desirable across different cultural settings (13).

The relevance of the cultural relativity of the QoL concept in LMICs is of great magnitude. While QoL has long been an explicit or implicit policy goal for international aid and development programmes (15), tracking the effectiveness of such programmes has relied on high order composite indicators which were never intended for this purpose. These include the United Nations Human Development Index, the country's economic growth such as per capita gross domestic product, standard of living or the millennium development goals with some serious drawbacks (16). Challenges include the selective definition of goals, definition of targets that are hardly ambitious, one-dimensional definition of poverty as a determinant of poor QoL, methodical problems in measuring poverty, quantity ahead of quality, blindness towards distribution issues, and above all, the focus on the South accountability to the North without commensurate measurable goals for the North (16).

A QoL measure for use in development aid programmes is urgently needed. The application of QoL scales in resource-poor settings has been limited for several reasons. The KIDSCREEN, DISABKIDS, EQ-5D-Y, and the Pediatric Quality of Life Inventory PedsQL are available only in few European languages and are yet to be validated in resource-poor settings, thus cross-cultural research is precluded (17). Similarly, the appropriateness of the World Health Organization Quality of Life (WHOQOL-BREF) for use among youth has been questioned, as its development was almost based on adults or adult patients, and discussions about its applicability and/or validation in children and adolescents are scarce (18). Some authors have called for the development of a new adolescent version of the WHOQOL (19).

Notwithstanding its limitations, the WHOQOL-BREF has been administered in LMICs with mixed results. Some of the questions, mainly pertaining to sex life, pain, and medication dependence, have been found to be culturally inappropriate among youth in Taiwan, attracting significant missing data and/or poor performance (18). Likewise, the three items measuring ‘pain and discomfort’, ‘medication required in everyday life’, and ‘negative feelings like anxiety and depression’ performed poorly among Indian children and the author suggested that such items should be dropped in future research (20). The environmental domain performed poorly, scoring the lowest among youth in India (20) and China (21). In Bangladesh, the WHOQOL-BREF physical and social relationships had poor internal consistency, with Cronbach alpha of 0.59 and 0.28, respectively (22). Recently, Paltzer et al. (23) found that, of the seven instruments that measure health-related QoL of young children, six have been used in resource-limited settings. However, they found that no instruments have specifically been developed for measuring health-related QoL among young children in resource-limited settings.

Therefore, for most LMICs, customised QoL measures need to take into account the structural issues that negatively affect the QoL of the population including structural poverty characterised by the unequal distribution and acquisition of assets, culturally mediated social relations and support, deprivation and unemployment due to lack of skills and poor access to vocational training programmes, and poor access to health and social services. The current study sought to develop a QoL instrument that addresses these issues as part of the baseline survey for the ‘Urban Program on Livelihoods and Income Fortification and Socio-Civic Transformation for the Youth in Kampala Project’. Implemented by World Vision Uganda with the support from World Vision Australia and the Australian Government Department of Foreign Affairs and Trade, the aim of the project is to improve the QoL of 3,500 people aged 13–24 in two of the most disadvantaged and populous divisions of Kampala, Uganda. Therefore, the aim of the study was twofold: 1) to develop and validate QoL instrument appropriate to the Ugandan context and closely related to structural issues faced by youth and 2) to assess factors associated with QoL among youth in Kampala.

## Methodology

### Operational definition and study setting

The Constitution of Uganda defines a youth as any person aged between 18 and 30 years, whereas the Ugandan Ministry of Gender, Labour, and Social Development uses the ages of 12 through 30 years in its various youth programmes (24). In contrast, the International Labour Organization (ILO) defines a youth as any individual in the 15–24 year age bracket (25). Therefore, for the purpose of our study, we defined a youth as any person aged between 13 and 24 years to take into account the ILO's definition and the Ugandan contextual reality.

The study was carried out in Makindye and Nakawa Divisions of Kampala City, Uganda, which has one of the youngest and the fastest growing population in Africa, with its population estimated at 35.5 million. More than three quarters (78%) are aged below 30 years (26). Despite the Ugandan youth making up the largest proportion of the population, they are mostly unproductive, with youth unemployment rates of 61.6% among the highest in Africa (27, 28). The low employment opportunities outside the capital Kampala attract a huge number of youth to the city in search of work. Almost 46% of the 1,723,300 people living in the city of Kampala reside in Nakawa and Makindye (29), which are the two divisions in which the project is being implemented. Since the past two decades, the two divisions have been characterised by high unemployment in tandem with a high dependence burden and rampant crimes (30), which present strong barriers to youth participation in community programmes and negatively affect their well-being. The study was approved by the Monash University Human Research Ethics Committee (approval no. CF16/1001-2016000532).

### Study design, sampling strategy

A cross-sectional survey was carried out in July 2014. The Kampala City Council is composed of divisions, and each administrative division is made of parishes, divided into zones. A list of all zones within the parishes of the Makindye and Nakawa divisions was established for sampling purposes. Our sampling unit within a zone was a household. A list of households in each of the zones was constructed with the help of the local council representatives (known as LCs), and from the list, a household was selected using a systematic sampling approach.

Households in each zone were given a unique identification number. Given that zones varied in size, the number of households to be surveyed in each zone was proportional to each size. The sampling interval (X) was determined by dividing the total number of households in each zone with the expected sample size, and the first household to be surveyed was randomly selected by choosing a number between I and X. The next household to be visited was selected by adding X to the first randomly selected number and the process continued until the required sample size for that zone was obtained. Hence, for each selected household, a person aged 13–24 took part in the study, and the interview occurred outside the home away from the rest of household members. In the case of a household having more than one eligible participant, the interviewer randomly selected one participant to be included in the study. If the selected household was not inhabited or no one was at home, the closest neighbouring household was used for the survey.

### Sample size and data collection

Given that employment status is closely associated with QoL, the prevalence of unemployment was used to determine our sample size. While obtaining accurate data on unemployment among youth in Uganda is a daunting task, available data suggest that youth unemployment rates vary between 34 and 83% (31, 32). For the purpose of sample size calculation, a prevalence of 40% was used. With a total population base from which to draw our sample of approximately 793,000 (average household size=5 and % of people aged 13–24 years=30%) (33) accepting a margin of error of 5% with 99% confidence interval, we estimated that we would need to obtain data on 637 participants.

### Training enumerators and governance

Data were collected by 12 trained enumerators, who were supervised by four experienced field coordinators to monitor quality control. Data enumerators, who were bilingual (English and Luganda), were trained over 3 days followed by a field-testing of the questionnaire prior to data collection to ascertain its cultural appropriateness. The training covered sampling technics, interview techniques, and ethical issues including confidentiality and respect, and bilingual workers’ familiarisation with the questionnaire. Bilingual workers administered the survey in English. The research implementation was overseen by a steering committee comprising four staff from World Vision, four field coordinators, and representatives from youth organisations. The steering committee commented on the questionnaire including the fine-tuning of the questionnaire for clarity and cultural appropriateness, and approved all research processes.

### Measures

#### QoL

The QoL questionnaire included 36 questions divided into a two-part scale (Appendix 1). That is, 18 questions were rated for satisfaction (Part 1) and 18 other questions rated on importance (Part 2). The questionnaire used Likert-scaled responses as follows: *satisfaction:* 1=very dissatisfied, 2=dissatisfied, 3=neither dissatisfied nor satisfied, 4=satisfied, and 5=very satisfied; *importance*: 1=not at all important, 2=somewhat important, 3=important, 4=very important; and 5=indispensable. Five questions were adapted from the WHOQOL-BREF (20): 1) housing conditions, 2) health services, 3) transportation, 4) sex life, and 5) personal safety. Additional questions were formulated to address the collective nature of social relationships [i.e. 1) the number of friends young people have, 2) how they get along with their friends, 3) how they get along with their family, 4) how they get along with non-relatives they live with, and 5) how they get along with non-relatives they do not live with]; access to and control over financial resources [i.e. 1) the amount of money young people have and 2) the amount of control they have over their money]; structural poverty and deprivation [i.e. 1) the work opportunities/career options, 2) the neighbourhood environment, 3) access to food, and 4) clothing]; and the use of time [i.e. the way young people spend their time]. Finally, a single-item well-being measure was included to assess concurrent construct validity. The item asked participants to rank themselves on a well-being ladder as follows: ‘Assume that a ladder with 10 steps is a way of picturing your life. Step 1 and the bottom of the ladder means the worst possible life for you. Step 10 and the top of the ladder means the best possible life for you. On the ladder, which step do you feel represents where you currently are in life (please circle)’.

#### Demographic, socio-economic, and lifestyle factors

A series of questions were administered as part of the survey to obtain background information on each participant. Data were collected regarding child age (in years), child gender (coded 0=female, 1=male), educational attainment (in years), marital status (coded 0=single/never married, 1=married, 2=cohabitation, 3=divorced/separated, and 4=widow/widower), living structure (coded 0=living with mother and father, 1=living with mother only, 2=living with father only, 3=parents are alive but living alone, 4=parents not alive, living with relative, 5=parents not alive, living with non-relative families, 6=parents not alive, living with friends, and 7=my parents are alive but live with other relatives), monthly income (in Ugandan Shillings-UGX), whether they have a disability (coded 0=no and 1=yes), whether they have attended any vocational training (coded 0=no and 1=yes), and employment status (coded 0=paid employment on a salary, 1=self-employed, 2=still at school, 3=volunteering or doing unpaid work; 4=unemployed: no structured activities, and 5=other, mainly commission based). The study also collected data on lifestyle factors including drug and substance misuse. Participant answered yes or no on questions related to smoking cigar, cigarette, shisha, or marijuana; chewing khat; khuber or smokeless tobacco; taking heroin; taking sedatives or stimulants; and sniffing paint, petrol, or glue.

### Statistical analysis

The QoL *satisfaction* scales were subjected to exploratory factor analysis (EFA) to determine the factor structures and confirmatory factor analysis (CFA) to provide the evidence for the factor structures using Amos 21.0 (SPSS, Chicago, IL, US). The total sample was randomly divided into two split-half samples (Sample 1, *N*=310; Sample 2, *N*=353). Various researchers have suggested that using one sample for the EFA and the other sample for the CFA is sufficient to confirm both the reliability and goodness of fit of any theory-based measures using structural equation modelling (SEM) (34).

In the EFA for sample I, standard procedures of principal component analysis (PCA) with Varimax rotation was used to determine the factor structure. The number of factors retained was determined by the Scree test, and the factor was determined using the Eigen values greater than 1, and item loadings ≥0.4 on a factor were considered to be robustly associated with that factor and those with loading <0.4 were considered weak (35). The Kaiser–Meyer–Olkin (KMO) index was used to measure the homogeneity of variables and to check if the data were suited for factor analysis, with KMO >0.60 used as a cut-off point to confirm the suitability of factor analyses (36). Cronbach alpha was computed to assess the internal consistency reliability of the scales. The following cut-off points were used to interpret Cronbach alpha: *α*≥0.9=excellent, 0.7≤*α*< 0.9=good, 0.6≤*α*<0.7=acceptable, 0.5≤*α*≤0.6=poor, and *α*<0.5=unacceptable (37).

In the CFA for sample II, maximum likelihood estimation (38) was used to validate the factor structure. The following indices and associated cut-off points were used to evaluate the model and to indicate a good fit (39): the chi-square associated with each degree of freedom (CMIN/DF) <3, the Goodness of Fit (GFI), Comparative Fit Index (CFI), Normed Fit Index (NFI), and Tucker Lewis Index (TLI)≥0.90, Root Mean Square Error of Approximation (RMSEA)≤0.08, and p of Close Fit (PCLOSE) >0.05. Concurrent validity was determined using two approaches. First, the QoL factors were correlated between them. We expected a high correlation between QoL component scores. The second approach assessed concurrent validity by examining the correlation between QoL scores and scores of the one single-item ranking well-being on a ladder.

Once the factors were confirmed and their psychometric properties established based on the ‘satisfaction’ scale, they were weighted for importance rating (40). The subscale scores were weighted based on Ferrans and Powers’ algorism (41). The first stage involved weighting satisfaction scores at item levels. The satisfaction score was centred to zero by subtracting three from satisfaction responses for each item (and the scale became −2, −1, 0, +1, +2). The next stage involved multiplying centred satisfaction response with the paired raw importance response (scored 1–5) for each item, giving a possible range for scores from –10 to +10. Finally, to eliminate any negative values for the final score, we added 10 to every item score to produce a possible range from 0 to +20. In order to make the scores easy to interpret, the total weighted subscale scores, together with the single-item well-being measure, were rescaled to a 100 point scale, from 0 (poor QoL or poor well-being) to 100 (perfect QoL or well-being) using the following formula (40):

% of scale maximum=[(score − 1) × 100/( number of scale points−1)]

Prior to undertaking planned analyses, the distribution of scores on each satisfaction and importance scaling variable was assessed by calculating mean, standard deviation, skewness, and kurtosis values. The normal distribution of the data was established using Kline's (42) proposed cut-off points of <3.0 for skewedness and <8.0 for kurtosis. Correlations of item satisfaction and item importance, and the Akaike Information Criteria (AIC) and the Bayesian Information Criteria (BIC) were used to assess whether weighting for importance rating matters in the Ugandan context (43).

The ‘*fitstat*’ command in Stata was used to compute the AIC and BIC to compare models (weighted vs. unweighted for importance), with the larger difference in either AIC or BIC indicating stronger evidence for one model over the other (i.e. the better model has lower AIC and/or BIC values). The effect of demographic, socio-economic, and lifestyle factors on unweighted and weighted QoL scores was assessed using linear regressions. The univariate analyses were screened to identify variables to be included in the multiple linear analyses and all variables whose *p*-value approached significance at 10% underwent multiple regression analyses (44). However, the level of statistical significance for establishing an association was set at a probability of *p*<0.05 for all tests.

## Results

### Demographic characteristics

The demographic and socio-economic characteristics are summarised in Table 1 for each split-half sample. The two split-half samples did not differ significantly on the study variables, except for gender. The majority of the sample (81.1%) were single or never married, which is consistent with study's age target. Only a quarter of participants (24.9%) lived in a nuclear family, while 24.6% lived in a single-parent household and 23% lived alone. Only 20.3% of participants were in the workforce as employees and about one in ten (8.8%) youth reported drug and substance misuse (Table 1).

**Table 1:** Demographic characteristics of the two split-half samples and testing for differences in the study variables

	Total sample		Sample 1		Sample 2		
				
Characteristic	*N*	Statistics	*N*	Statistics	*N*	Statistics	*p*
All	663	100%	310	100%	353	100%	
Gender							0.045
Female	292	44.9%	123	40.7%	169	48.6%	
Male	358	55.1%	179	59.3%	179	51.4%	
Age in years (mean±SD)	657	19.5±3.5	306	19.5±3.4	351	19.5±3.6	0.965
Monthly income (UGX in 10,000; mean±SD)	621	154±27	290	173±34	331	137±20	0.099
Disability							0.984
Yes	49	7.5%	23	7.5%	26	7.4%	
No	607	92.5%	284	92.5%	323	92.6%	
Level of education in years (mean±SD)	659	9.2±4.0	308	9.1 (3.9)	351	9.2 (4.0)	0.880
Marital status							0.730
Single/never married	528	81.1%	241	79.8%	287	82.2%	
Married	49	7.5%	23	7.6%	26	7.5%	
Cohabitation	55	8.5%	27	9.0%	28	8.0%	
Divorced/separated	19	2.9%	11	3.6%	8	2.3%	
Living structure							0.106
Mother and father	152	24.9%	79	27.8%	73	22.4%	
Mother only	123	20.2%	60	21.1%	63	19.3%	
Father only	27	4.4%	9	3.2%	18	5.5%	
Alone, but parents are alive	140	23.0%	64	22.5%	76	23.3%	
Relative, parents not alive	27	4.4%	8	2.8%	19	5.8%	
Non-relative families, parents not alive	16	2.6%	10	3.5%	6	1.8%	
Friends, parents not alive	27	4.4%	8	2.8%	19	5.8%	
Other relatives, but parents alive	98	16.1%	46	16.2%	52	16.0%	
Attended any vocational or technical training							0.755
No	481	78.2%	226	77.7%	255	78.7%	
Yes	134	21.8%	65	22.3%	69	21.3%	
Employment status							0.689
Paid employment on a salary	132	20.3%	63	20.9%	69	19.8%	
Self-employed	165	25.4%	69	22.9%	96	27.6%	
Still at school	190	29.2%	87	28.8%	103	29.6%	
Volunteering or doing unpaid work	33	5.1%	18	6.0%	15	4.3%	
Unemployed: no structured activities	116	17.9%	58	19.2%	58	16.7%	
Other, mainly commission based	14	2.2%	7	2.3%	7	2.0%	
Drug and substance misuse							0.158
No	605	91.2%	288	92.9%	317	89.8%	
Yes	58	8.8%	22	7.1%	36	10.2%	

### Construct validity and reliability

The KMO coefficient was 0.90 and Bartlett's Sphericity test was found to be significant =2475.9, *p*<0.001), indicating factor analyses was justified. Data from the EFA are summarised in Table 2. The principal component factor analysis of the 18 items on *satisfaction* with Varimax rotation method yielded three factors: living conditions and lifestyle (seven items, *α*=0.84), social relationships (five items, *α*=0.86), and personal independence (five items, *α*=0.76). The items loaded well on their respective subscales (a minimum item loading of 0.51), with Eigen values greater than 1 (6.95 for living conditions and lifestyle, 1.89 for social relationships, and 1.25 for personal independence). The extraction of the two factors accounted for 56% of the total variance. However, the item ‘How satisfied are you with your neighbourhood as a place to live in?’ cross-loaded on all three factors (loading of 0.31 for factor 1, 0.36 for factor 2, and 0.38 for factor 3) and was subsequently deleted.

**Table 2:** Factor loadings, item means, standard deviation (SD), and Cronbach alpha coefficients for the two split-half samples

	Sample 1 *N*=310		Sample 2 *N*=353	
		
	EFA loading	Mean (SD)	CFA loading	Mean (SD)
Factor 1: Living conditions and lifestyle	(α=0.84)		(α=0.85)	
The clothing you wear?	0.75	3.66 (1.09)	0.82	3.67 (1.04)
The food you eat?	0.71	3.74 (1.05)	0.79	3.75 (0.97)
Your sex life?	0.67	3.77 (0.93)	0.50	3.72 (0.98)
Your personal safety?	0.67	3.57 (1.02)	0.76	3.57 (1.07)
The health services you use?	0.65	3.36 (1.19)	0.72	3.44 (1.12)
Your access to transportation?	0.62	3.53 (1.08)	0.66	3.55 (1.04)
The way you spend your time?	0.52	3.64 (1.01)	0.45	3.65 (0.99)
Factor 2: Social relationships	(α=0.86)		(α=0.85)	
How you get along with your friends?	0.85	3.80 (0.90)	0.79	3.83 (0.85)
The people with whom you live?	0.82	3.91 (0.83)	0.70	3.88 (0.86)
The number of friends you have?	0.79	3.74 (1.01)	0.62	3.78 (0.94)
How you get along with other people in the community?	0.75	3.76 (0.98)	0.74	3.80 (0.90)
Your relationship with all the other people in your family?	0.56	3.96 (0.99)	0.78	3.92 (0.96)
Factor 3: Personal independence	(α=0.76)		(α=0.72)	
The amount of money you have?	0.78	2.59 (1.36)	0.54	2.56 (1.34)
The work opportunities available to you?	0.72	2.71 (1.36)	0.61	2.87 (1.37)
Free time you have to be alone without any worry?	0.61	3.11 (1.17)	0.48	3.55 (1.15)
With your current dwelling?	0.52	3.39 (1.14)	0.70	3.50 (1.08)
The amount of control you have over your money	0.51	3.42 (1.20)	0.57	3.41 (1.24)

CFA=confirmatory factor analysis; EFA=exploratory factor analysis.

**Fig. 1:**
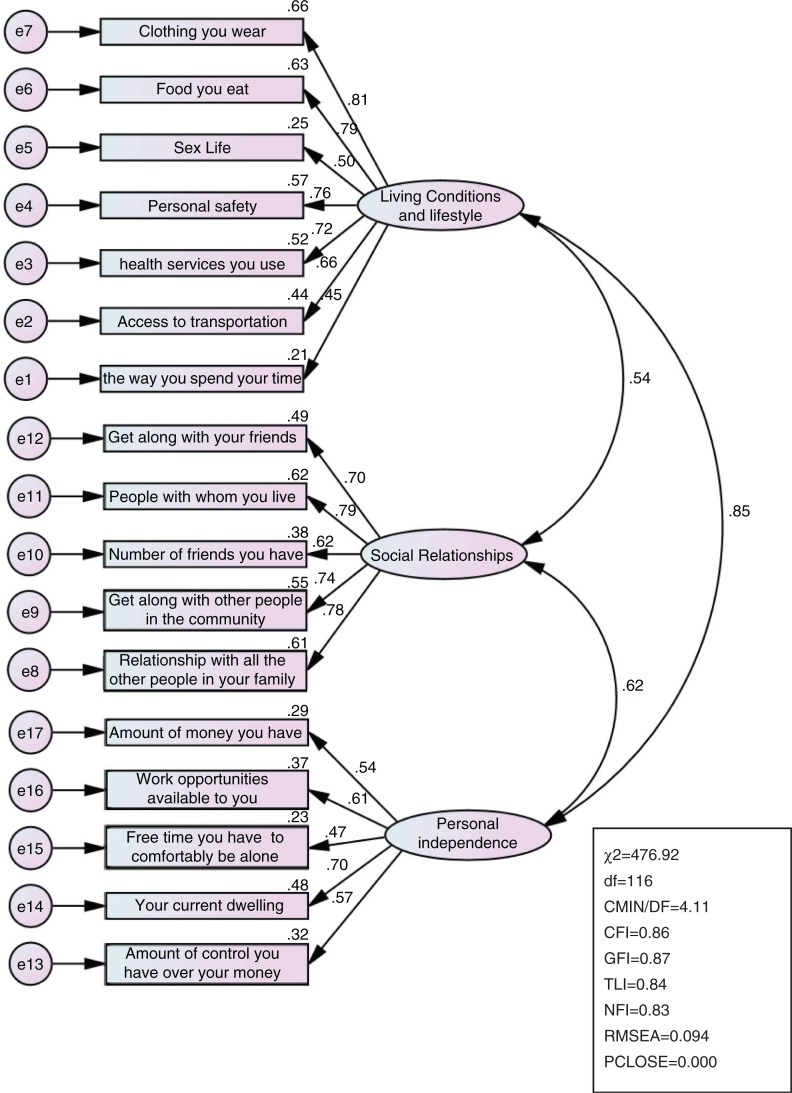
Confirmatory factor analyses: initial non-respecified model.

**Fig. 2:**
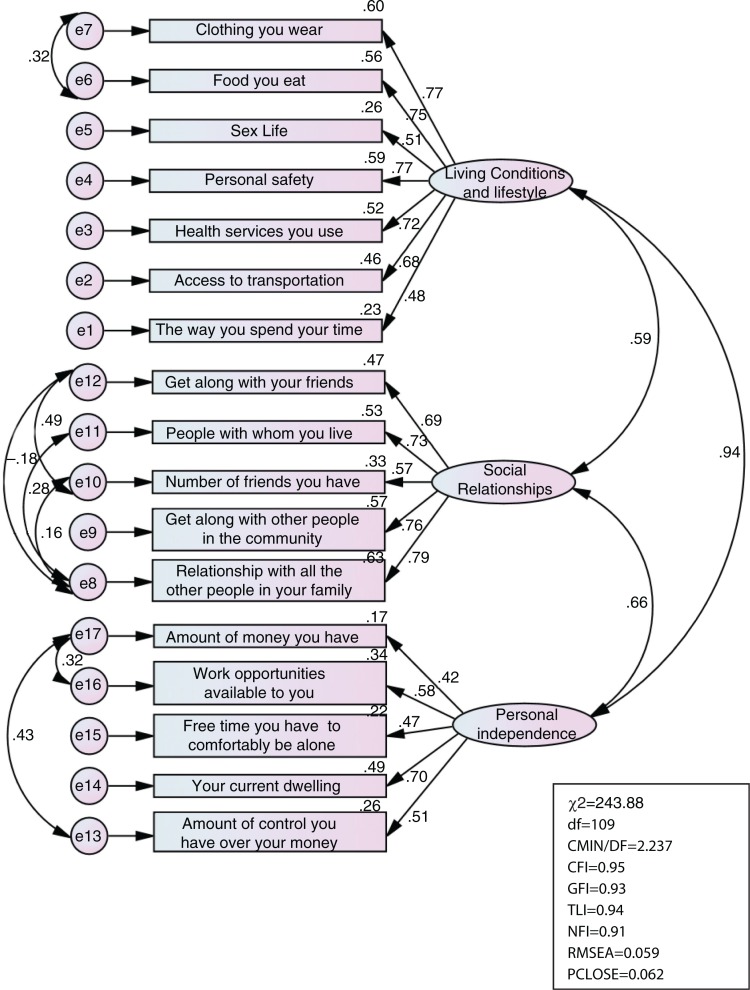
Confirmatory factor analyses: respecified model.

Data from the CFA are summarised in Figs. 1 and 2. The findings suggest that the initial model (Fig. 1) demonstrated a poor to marginal fit model. The initial model was respecified by examining modification indices (Fig. 1), resulting in a good model fit: CMIN/DF=2.24, CFI=0.95, TLI=0.94; NFI=0.91; RMSEA=0.06; SRMR=0.027, PCLOSE>0.05. The single-item ranking well-being on the ladder was also highly correlated with the QoL components, hence demonstrating a strong concurrent validity. It had a correlation coefficient 0.21 (*p*<0.001) with living conditions and lifestyle, 0.30 (*p*<0.001) with social relationships, and 0.11 (*p*<0.01) with personal independence. The three QoL components were also highly correlated: living conditions and lifestyle was highly correlated with social relationships (*r*=0.65, *p*<0.0010) and personal independence (*r*=0.50, *p*<0.001), while personal independence was highly correlated with social relationship ships (*r*=0.48, *p*<0.001).

### Data distribution

Data presented in Tables 3 and 4 suggest that the skewness and kurtosis values for individual satisfaction items varied from −1.28 to 1.35 and −0.52 to 5.02, respectively. For individual importance items, the values varied from −1.06 to −0.61 for skewedness and 2.73 to 4.46 for kurtosis. The computed subscales had values varying from −1.16 to 0.73 for skewness and 2.59 to 5.11 for kurtosis (Table 4). These values indicate that there was no evidence of univariate non-normality and parametric testing was justified.

**Table 3:** Means, standard deviations (SD), skewness, and kurtosis for satisfaction and importance ratings on each item and correlation between satisfaction and importance items (*N*=663)

	Satisfaction				Importance				Weighted scores		
				
Items	Mean	SD	Skewness	Kurtosis	Mean	SD	Skewness	Kurtosis	Mean	SD	Correlation^a^
Living conditions and lifestyle											
The clothing you wear?	3.68	1.05	−0.93	3.25	3.68	0.91	−0.77	3.59	12.89	3.99	0.45***
The food you eat?	3.76	1.00	−1.07	3.75	3.74	0.88	−0.86	4.00	13.19	3.87	0.46***
Your sex life?	3.78	0.92	−0.85	3.46	3.65	1.01	−0.85	3.32	13.00	3.79	0.31***
Your personal safety?	3.58	1.04	−0.75	2.89	3.84	0.89	−0.78	3.66	12.60	4.11	0.43***
The health services you use?	3.42	1.15	−0.57	2.43	3.74	0.90	−0.74	3.49	11.85	4.55	0.32***
Your access to transportation?	3.57	1.04	−0.80	3.00	3.66	0.91	−0.91	3.86	12.34	4.00	0.38***
The way you spend your time?	3.67	0.98	−1.10	3.70	3.67	0.89	−0.87	3.73	12.77	3.60	0.45***
Social relationships											
How you get along with your friends?	3.83	0.86	−1.28	5.02	3.76	0.84	−0.97	4.46	13.40	3.33	0.47***
The people with whom you live?	3.92	0.83	−1.09	4.58	3.79	0.86	−0.86	4.01	13.72	3.45	0.45**
The number of friends you have?	3.78	0.96	−1.13	4.08	3.68	0.92	−0.99	4.05	13.13	3.64	0.38***
Getting along with other people in the community?	3.80	0.93	−1.06	4.23	3.72	0.90	−0.91	4.08	13.26	3.67	0.42***
Your relationship with other people in your family?	3.95	0.96	−1.20	4.38	3.88	0.86	−0.87	4.10	14.08	3.90	0.52***
Personal independence											
The amount of money you have?	2.58	1.35	0.33	1.80	3.84	0.95	−1.06	4.23	8.70	5.44	0.27***
The work opportunities available to you?	2.81	1.38	0.05	1.62	3.78	0.98	−0.84	3.49	9.53	5.38	0.23***
Free time you have to be alone without any worry?	3.26	1.16	1.35	−0.52	3.39	1.09	−0.61	2.73	11.42	3.99	0.48***
With your current dwelling?	3.47	1.10	−0.80	2.76	3.65	0.91	−0.92	3.96	12.08	4.06	0.43***
The amount of control you have over your money?	3.43	1.21	−0.60	2.35	3.79	0.91	−1.02	4.38	12.09	4.74	0.48***

a Correlations between item satisfaction and item importance.

* *p*<0.05

** *p*<0.01

*** *p*<0.001

**Table 4:** Minimum, maximum, means, standard deviations (SD), skewness, and kurtosis for derived subscales (*N*=663)

Subscale	Minimum	Maximum	Mean	SD	Skewness	Kurtosis
Living conditions and lifestyle (7 items)						
Raw and unscaled	7	35	25.31	5.24	−0.73	3.56
Rescaled but unweighted for importance	0	100	65.41	18.74	0.73	3.56
Rescaled and weighted for importance	0	100	53.82	16.46	0.16	2.84
Social relationships (5 items)						
Raw and unscaled	5	25	19.19	3.66	−1.16	5.11
Rescaled but unweighted for importance	0	100	70.94	18.31	−1.16	5.11
Rescaled and weighted for importance	0	100	64.00	15.92	−0.17	3.56
Personal independence (5 items)						
Raw and unscaled	5	25	15.47	4.37	−0.29	2.59
Rescaled but unweighted for importance	0	100	52.33	21.86	−0.29	2.59
Rescaled and weighted for importance	0	100	58.97	17.95	−0.02	2.96

### Relevance of incorporating perceived importance into the quality of life measure

Correlations of item satisfaction and item importance are summarised in Tables 3 and 4, which were all significant at *p*<0.001 and ranged from 0.23 to 0.52. The AIC and BIC results are summarised in Table 5. The model incorporating perceived importance had lower AIC and BIC values than the unweighted model, hence providing very strong support for the weighted model. The univariate linear regression analyses (unweighted vs. weighted satisfaction scores) examining the effect of demographic, socio-economic, and lifestyle factors on QoL are summarised in Appendix 1.

**Table 5:** Results for model comparison between weighted and unweighted for importance

	Living conditions and lifestyle			Social relationships			Personal independence		
			
Parameters	Weighted model (A)	Unweighted model (B)	Difference (A–B)	Weighted model (A)	Unweighted model (B)	Difference (A–B)	Weighted model (A)	Unweighted model (B)	Difference (A−B)
Log-likelihood									
Intercept only	−2258.415	−2266.742	8.327	−2174.316	−2230.414	56.099	−2220.829	−2360.524	139.695
Full model	−2227.716	−2230.964	3.248	−2148.154	−2204.064	55.91	−2202.666	−2333.997	131.332
AIC	8.546	8.559	−0.012	8.244	8.457	−0.213	8.451	8.951	−0.499
BIC	1285.189	1291.686	−6.497	1126.066	1237.886	−111.821	1235.089	1497.752	−262.663

AIC=Akaike Information Criterion; BIC=Bayesian Information Criteria; A=current model; B=saved model.

The multiple linear regression analyses are summarised in Table 6 using the unweighted scores in the regression on living conditions and lifestyle, the effect of disability, marital status, and employment, which were significant in the univariate linear regression analyses (Appendix 2), became non-significant. However, the effect of drug and substance use, family structure, and educational attainment remained significant. That is, poor living condition and lifestyle remained consistently and significantly associated with drug and substance misuse (*β*=−11.78, *p*<0.001), living with a single mother (*β*=−8.36, *p*<0.001), and leaving home to live alone (*β*=−6.99, *p*<0.01). In contrast, living conditions and lifestyle scores were positively associated with educational attainment (β=0.61, *p*<0.01). When living conditions and lifestyle scores weighted for perceived importance were used, the pattern was more robust, with marital status and being an orphan remaining significant as they were in the univariate model, hence providing evidence of the advantage for using weighted scores.

**Table 6:** Multivariate analyses of the effect of demographic, socio-economic, and lifestyle factors on quality of life: rescaled, unweighted satisfaction scores vs. rescaled satisfaction scores, weighted for importance

	Rescaled, unweighted satisfaction scores Aβ (95% CI)			Rescaled satisfaction scores, weighted for importance A (95% CI)		
		
	Living conditions and lifestyle	Social relationships	Personal independence	Living conditions and lifestyle	Social relationships	Personal independence
Gender, male	0.43 (−2.65, 3.50)	2.31 (−0.62, 5.23)	0.73 (−3.00, 4.47)	0.85 (−2.21, 3.91)	2.50 (−0.13, 5.13)	1.03 (−1.88, 3.94)
Age in years	−0.05 (−0.71, 0.61)	−0.16 (−0.79, 0.46)	0.32 (−0.48, 1.12)	−0.07 (−0.73, 0.59)	−0.17 (−0.74, 0.39)	0.10 (−0.52, 0.73)
Monthly income	0.00 (−0.06, 0.06)	−0.01 (−0.06, 0.05)	0.02 (−0.05, 0.09)	0.00 (−0.06, 0.06)	0.00 (−0.05, 0.05)	0.01 (−0.04, 0.07)
Disability, yes	−3.76 (−9.61, 2.09)	−2.99 (−8.55, 2.57)	−5.01 (−12.12, 2.09)	−2.01 (−7.83, 3.82)	−2.34 (−7.34, 2.67)	−1.40 (−6.94, 4.13)
Drug and substance misuse, yes	−11.78*** (−17.13, −6.43)	−8.53*** (−13.62, −3.45)	−4.67 (−11.17, 1.83)	−10.89** (−16.22, −5.56)	−8.18*** (12.76, −3.60)	−3.17 (−8.24, 1.89)
Level of education in years	0.61** (0.11, 1.12)	0.65** (0.16, 1.13)	0.47 (−0.14, 1.09)	0.55* (0.04, 1.05)	0.60*** (0.17, 1.04)	0.35 (−0.13, 0.83)
Marital status (Ref=single/never married)						
Married	6.58 (−0.02, 13.18)	5.16 (−0.05, 10.37)	5.38 (−2.47, 13.24)	6.50 (−0.04, 13.04)	6.33 (−0.01, 12.67)	3.73 (−2.40, 9.85)
Cohabitation	6.15 (−0.47, 12.76)	3.45 (−2.83, 9.74)	8.11* (0.07, 16.14)	7.07* (0.48, 13.65)	2.09 (−3.57, 7.75)	6.33 (−0.05, 12.71)
Divorced/separated	7.05 (−2.75, 16.84)	8.49 (−0.82, 17.8)	6.46 (−5.43, 18.35)	6.48 (−3.27, 16.22)	8.66 (−0.03, 17.35)	6.60 (−2.66, 15.87)
Family structure (Ref=nuclear family): live with						
Mother only	−8.36*** (12.92, −3.90)	−4.70* (−9.03,−0.36)	−7.35** (−12.89, −1.81)	−7.52*** (−12.07,−2.98)	−3.96* (−7.86, −0.05)	−4.57* (−8.89,−0.25)
Father only	−3.84 (−11.26, 3.58)	−1.47 (−8.52, 5.59)	−4.03 (−13.04, 4.99)	−4.64 (−12.03, 2.75)	−3.00 (−9.35, 3.36)	−2.99 (−10.02, 4.04)
Alone, but parents are alive	−6.99** (−11.83,−2.16)	−5.39* (−9.98, −0.80)	−6.34 (−12.75, 0.07)	−7.08*** (−11.89, −2.26)	−5.38** (−9.51, −1.24)	−4.49 (−9.06, 0.09)
Relatives, parents not alive	2.21 (−5.49, 9.91)	−2.18 (−9.49, 5.14)	3.82 (−5.53, 13.16)	1.68 (−5.98, 9.34)	−2.38 (−8.96, 4.21)	2.42 (−4.86, 9.71)
Non-relatives, parents not alive	−9.37 (−18.94, 0.21)	0.92 (−8.18, 10.01)	−9.01 (−20.64, 2.63)	−11.09* (−20.62, −1.550	0.11 (−8.09, 8.31)	−6.41 (−15.48, 2.65)
Fiends, parents not alive	−6.83 (−15.21, 1.55)	3.14 (−4.82, 11.10)	−6.96 (−17.13, 3.22)	−7.70 (−16.04, 0.64)	3.09 (−4.08, 10.26)	−5.48 (−13.42, 2.45)
Other relatives, but parents alive	−4.11 (−9.00, 0.77)	−3.30 (−7.94, 1.33)	−3.59 (−9.52, 2.34)	−4.33 (−9.19, 0.53)	−2.87 (−7.05, 1.31)	−2.66 (−7.28, 1.96)
Employment status (Ref=employed)						
Self-employed	1.72 (−2.82, 6.25)	−1.07 (−5.38, 3.24)	1.18 (−4.33, 6.69)	1.82 (−2.70, 6.34)	−1.22 (−5.10, 2.67)	0.91 (−3.38, 5.21)
Still at school	−2.08 (−6.93, 2.77)	0.82 (−3.79, 5.43)	−2.07 (−7.96, 3.82)	−2.04 (−6.87, 2.79)	−0.30 (−4.45, 3.85)	−0.63 (−5.22, 3.96)
Volunteering or doing unpaid work	−3.46 (−11.21, 4.29)	1.54 (−5.82, 8.90)	0.00 (−9.41, 9.41)	−2.77 (−10.49, 4.95)	1.09 (−5.55, 7.72)	−0.34 (−7.67, 7.00)
Unemployed: no structured activities	−4.43 (−9.52, 0.66)	−4.69 (−9.43, −0.05)	−7.08* (−13.26, −0.89)	−2.95 (−8.02, 2.12)	−3.04 (−7.40, 1.32)	−3.42 (−8.24, 1.40)
Other, mainly commission based	6.14 (−5.16, 17.45)	1.72 (−9.02, 12.46)	−17.31* (−31.05, −3.58)	4.19 (−7.07, 15.45)	0.17 (−9.50, 9.85)	−13.35*** (−24.05, −2.64)

Aβ=adjusted regression coefficients; CI: confidence interval.

* *p*<0.05

** *p*<0.01

*** *p*<0.001.

Note: Adjusted for factors in the table.

In terms of social relationships, the multiple linear regression analyses (Table 6) found that the effect of disability using both unweighted and weighted scores and unemployment status using the weighted scores, which were significant in the univariate linear regression analyses (Appendix 2), became non-significant. However, the effect of educational attainment, drug and substance use, and family structure remained consistent. That is, in the regression on unweighted scores, poor social relationships were negatively associated with drug and substance misuse (*β*=−8.53, *p*<0.001), living with a single mother (*β*=−4.70, *p*<0.05), and running away from home to live alone (*β*=−5.39, *p*<0.05), while educational attainment was positively associated with social relationships scores. This pattern remained consistent in the regressions on the scores weighted for importance (Table 6).

In terms of personal independence, the multiple linear regression analyses (Table 6) found that the effect of age, monthly income, educational attainment, and family structure, which were significant in the univariate linear regression analyses (Appendix 2), became non-significant. However, personal independence scores were consistently positively associated with being in a cohabiting relationship (β=8.11, *p*<0.01), but low personal independence was significantly associated with living with a single mother (β=−7.35, *p*<0.01), volunteering or undertaking unpaid work as the sole mode of employment (β=−7.08, *p*<0.05), and being unemployed (β=−17.31, *p*<0.05). The regression on scores weighted from importance (Table 6) produced a similar pattern except for marital status, which became insignificant.

## Discussion

The aim of the study was to develop and validate a QoL instrument for use among youth and appropriate to the Ugandan context, and to assess factors associated with QoL among youth in Kampala. Our findings support a good model fit with strong concurrent validity, and suggest that using satisfaction scores weighted for importance rating provides a more robust and extended pattern of the relationship between living conditions and lifestyle and health determinants than unweighted scores. The need to weight satisfaction scores with importance ratings is further supported by the strong correlations between item satisfaction and item importance and AIC and BIC values. While our findings support Hsieh's (11, 12) overall findings, they do not support those suggesting non-significant correlations between item satisfaction and item importance (4, 5, 7, 45). Interestingly, none of these studies reported models fit, hence underscoring the novelty of our findings.

These inconsistent results in the literature could be explained by three major factors: failing to test for model fit, the cultural relativity of the QoL concept, and methodological differences. Most of the studies described above were undertaken in various settings where there exist cultural differences in values attached to specific social aspects of life, traditions, societal standards as well as customs. Existing methods of measuring QoL may not be adequate to capture important differences across cultures, especially as people may conceptualise QoL facets by drawing from their lived experiences and cultural norms (46). For example, Heady and Wearing (47) found that Northern Europeans (Danes, Swedes, Swiss, Norwegians), Dutch, Irish, and Australians reported higher levels of well-being than Japanese, Greeks, Italians, Spanish, and French. The authors attributed these differences to variations in cultural norms, equality, the level of democracy, and affluence. Similarly, measures of QoL across cultures have not used the same items, used different population (sick vs. healthy participants), and used different analytical approaches (5, 9–12, 19, 45, 48) making difficult to compare studies and results. Notwithstanding these limitations, our study demonstrate that the need to weight satisfactions scores with importance rating needs to be context specific to account for cultural influences and contextual backgrounds. The study also demonstrates that it is possible to incorporate QoL measure in development aid programmes.

We found that QoL among youth in Kampala was positively associated with educational attainment but negatively associated with employment status. The universal primary and secondary education policies in Uganda continue to increase access to education, (49, 50) but there is a mismatch between the skills youth are attaining at school and requirements in the job market. The Ugandan education system is theoretical, with little practice (51). Therefore, youth with low educational attainment find it difficult to find a job, and if they get employed they found themselves in low-quality, informal, insecure, and casual jobs with unstable wages. Employers prefer graduates of ordinary level or high school because they are affordable, do not demand higher salaries, can do the work with minimum training, and are quick learners that adapt to any work, and above all can tolerate unfavourable employment conditions. All these factors combine to affect their QoL and well-being. It is possible that high educational attainment in Uganda is associated with non-alienated work, better economic resources, and high personal control, factors known to be associated with better QoL (52).

Our findings suggest a close relationship between marital status and QoL. Cohabitation was associated with better QoL than being single/never married. The companionship provides a sense of security and emotional satisfaction, better financial security, and shared responsibilities. These findings are consistent with the literature suggesting that individuals in cohabiting relationships have better QoL and well-being than single/never married individuals (53).

We found that poor QoL was associated with drug and substance misuse among youth, which is consistent with the current literature. For example, Zullig and colleagues (54) examined the relationship between perceived life satisfaction and selected substance use behaviours among high school students. They found that factors significantly associated with reduced life satisfaction were cigarette smoking, chewing tobacco, marijuana, cocaine, regular alcohol use, binge drinking, injection drug, and steroid use. Similarly, Topolski et al. (55) explored the association between health-risk behaviours and self-perceived QoL among adolescents. They found that adolescent who engaged in one or multiple risk behaviours reported poorer QoL than abstainers.

We found that poor QoL was associated with living with a single mother and running away from home. Research from Africa suggests that single-parent families, especially single-mother-headed families, experience extreme poverty, exacerbated by inadequate public assistance, lower earning capacity, and lack of principal care subsidies which predispose them to a number of stressors (psychological difficulties, inadequate childcare, and social and financial stress) (56, 57). The consequence of such stress is the downward spiral of family disintegration (56, 57). Similarly, regardless of the family structure, a significant number of children run away from home to escape poverty, family-level child labour and abuse, family problems, and child neglect to live on the street and/or with friends (58). Therefore, coping with the circumstances of family poverty and disruption affects the QoL of the affected families, especially as children from single-mother-headed families and those running away from home. They are more likely drop out of school, to be idle out of school as well as work, to use drug and other illicit substances, and to engage in juvenile delinquency than those from nuclear families (59).

## Conclusions

The study provides evidence of the reliability and validity of the QoL tool which can be used to influence decisions in international aid and development interventions in resource-poor settings. It presents a number of opportunities to test theories of change to improve the effectiveness of international aid and development programmes in improving QoL. Currently, World Vision Uganda is using the tool in development programmes among youth in Kampala, to monitor the effectiveness of the ‘Urban Program on Livelihoods and Income Fortification and Socio-civic Transformation for the Youth in Kampala Project (UPLIFT)’ in improving QoL among youth, with the view of scaling it up across its programmes. The study methodology and findings demystify the measurement of the QoL in poor resourced areas with similar characteristics and also demonstrate that international aid and development programmes that seek to improve the QoL can confidently identify and attribute their contribution.

## Paper context

- Research on quality of life (QoL) has grown exponentially over the last four decades. However, existing instruments have predominantly focused on health-related dimensions in industrialised countries.
- While QoL has long been an explicit policy goal for international development aid programmes, no instruments have specifically been developed for measuring the effectiveness of international development aid programmes.
- The current study developed and validated a QoL instrument for use in international aid and development programmes. Our findings provide very strong support to weight satisfaction scores with importance ratings when measuring QoL in Uganda.
- This validated QoL measure will allow government and non-government organisations in LMICs to track progress of international aid and development programme.

## Appendix 1

### The Ugandan Youth Quality of Life index

Overall Life Satisfaction: single item to measure overall quality of life.

Assume that a ladder with 10 steps is a way of picturing your life (10-point Likert scale). Consider these steps represent a scale from 1 to 10 where step 1 represents the bottom of the ladder and means the worst possible life for you and step 10 represents top of the ladder and means the best possible life for you. On the ladder, which step do you feel represents where you currently are in life (please circle) …

**Table:** How satisfied are you with:

	Very dissatisfied	Dissatisfied	Neither dissatisfied nor satisfied	Satisfied	Very satisfied
The clothing you wear?	1	2	3	4	5
The food you eat?	1	2	3	4	5
Your sex life?	1	2	3	4	5
Your personal safety?	1	2	3	4	5
The health services you use?	1	2	3	4	5
Your access to transportation?	1	2	3	4	5
The way you spend your time?	1	2	3	4	5
How you get along with your friends	1	2	3	4	5
The people with whom you live?	1	2	3	4	5
The number of friends you have?	1	2	3	4	5
How you get along with other people in the community?	1	2	3	4	5
Your neighbourhood as a place to live in?	1	2	3	4	5
Your relationship with all the other people in your family?	1	2	3	4	5
The amount of money you have?	1	2	3	4	5
The work opportunities available to you?	1	2	3	4	5
The free time you have to comfortably be alone without any worry?	1	2	3	4	5
With your current dwelling?	1	2	3	4	5
The amount of control you have over your money	1	2	3	4	5

**Table:** How important to you:

	Not at all important	Somehow important	Important	Very important	Indispensable
Is the way you spend your time?	1	2	3	4	5
Is the clothing you wear?	1	2	3	4	5
Is the food you eat?	1	2	3	4	5
Is your sex life?	1	2	3	4	5
Is your personal safety?	1	2	3	4	5
Is the health services you use?	1	2	3	4	5
Is your access to transportation?	1	2	3	4	5
Is to get along with your friends?	1	2	3	4	5
Are the people with whom you live?	1	2	3	4	5
Is the number of friends you have?	1	2	3	4	5
Is your relationship with all the other people in your family?	1	2	3	4	5
Is to get along with other people in the community?	1	2	3	4	5
Is to have a good neighbourhood?	1	2	3	4	5
Is the amount of money you have?	1	2	3	4	5
Is the work opportunities available to you?	1	2	3	4	5
Is to feel comfortable when alone?	1	2	3	4	5
Is to have adequate housing?	1	2	3	4	5
Is the amount of control you have over your money?	1	2	3	4	5
					

## Appendix 2

**Table:** Univariate analyses of the effect of demographic, socio-economic, and lifestyle factors on quality of life: rescaled, unweighted satisfaction scores vs. rescaled satisfaction scores, weighted for importance

	Rescaled, unweighted satisfaction scores U (95% CI)			Rescaled satisfaction scores, weighted for importance U (95% CI)		
		
	Living conditions and lifestyle	Social relationships	Personal independence	Living conditions and lifestyle	Social relationships	Personal independence
Gender, male	−0.11 (−2.97, 2.74)	1.38 (−1.41, 4.16)	1.90 (−1.44, 5.25)	−0.12 (−2.89, 2.65)	1.39 (−1.34, 2.50)	1.58 (−0.95, 4.12)
Age in years	0.31 (−0.09, 0.72)	0.08 (−0.32, 0.47)	0.68** (0.21, 1.16)	0.22 (−0.17, 0.61)	0.03 (−0.31, 0.38)	0.32 (−0.04, 0.68)
Monthly income	0.04 (−0.01, 0.09)	0.02 (−0.03, 0.07)	0.08* (0.01, 0.14)	0.03 (−0.02, 0.08)	0.02 (−0.02, 0.07)	0.05* (0.00, 0.10)
Disability, yes	−7.41** (−12.79, −2.04)	−8.80*** (−14.02, −3.58)	−10.28*** (−16.61, −3.95)	−4.47 (−9.67, 0.72)	−6.96*** (−11.55, −2.37)	−5.08* (−9.88, −0.28)
Drug and substance misuse, yes	−12.70*** (−17.67, −7.73)	−9.54*** (−14.43, −4.64)	−5.77 (−11.65, 0.12)	−10.50*** (−15.28, −5.72)	−7.98*** (−12.23, −3.72)	−2.88 (−7.32, 1.56)
Level of education in years	0.68** (0.33, 1.04)	0.64*** (0.29, 0.99)	0.90*** (0.48, 1.32)	0.55*** (0.21, 0.90)	0.48*** (0.17, 0.78)	0.54** (0.23, 0.86)
**Marital status (Ref=single/never married)**						
Married	0.81 (−4.65, 6.27)	3.62 (−1.75, 8.98)	2.21 (−4.18, 8.59)	0.56 (−4.69, 5.80)	3.04 (−1.64, 7.72)	0.76 (−4.07, 5.59)
Cohabitation	6.18* (0.99, 11.36)	2.63 (−2.46, 7.42)	6.14* (0.08, 12.20)	6.20* (1.22, 11.17)	1.92 (−2.52, 6.36)	3.85 (−0.74, 8.43)
Divorced/separated	−1.51 (−10.06, 7.03)	1.38 (−7.01, 9.77)	0.86 (−9.12, 10.84)	0.35 (−7.85, 8.55)	3.05 (−4.26, 10.37)	2.86 (−4.70, 10.41)
**Family structure (Ref=nuclear family): live with**						
Mother only	−9.42*** (−13.83, −5.01)	−6.83** (−11.14, −2.52)	−9.32*** (−14.54, −4.09)	−8.38*** (−12.60, −4.17)	−5.46*** (−9.17, −1.75)	−5.42** (−9.37, −1.47)
Father only	−4.65 (−12.24, 2.95)	−2.73 (−10.16, 4.70)	−3.90 (−12.90, 5.10)	−5.52 (−12.78, 1.74)	−3.71 (−10.09, 2.68)	−2.85 (−9.65, 3.95)
Alone, but parents are alive	−6.19** (−10.45, −1.93)	−8.12*** (−12.29, −3.95)	−2.78 (−7.83, 2.26)	−5.99** (−10.06, −1.92)	−7.27*** (−10.85, −3.69)	−2.19 (−6.00, 1.63)
Relatives, parents not alive	−0.42 (−8.01, 7.18)	−7.36 (−14.79, 0.07)	1.10 (−7.90, 10.10)	−0.90 (−8.16, 6.36)	−6.83* (−13.22, −0.45)	0.19 (−6.61, 6.99)
Non-relatives, parents not alive	−11.72* (−21.28, −2.17)	−4.44 (−13.79, 4.91)	−11.05 (−22.38, 0.27)	−13.15** (−22.28, −4.01)	−4.76 (−12.80, 3.27)	−7.62 (−16.18, 0.94)
Friends, parents not alive	−13.11** (−20.71, −5.52)	−9.39* (−16.82, −1.97)	−13.16** (−22.16, −4.16)	−11.98*** (−19.23, −4.72)	−6.71* (−13.10, −0.32)	−8.55** (−15.35, −1.75)
Other relatives, but parents alive	−4.95* (−9.66, −0.24)	−6.20*** (−10.81, −1.59)	−4.48 (−10.06, 1.10)	−4.99* (−9.49, −0.48)	−5.21*** (−9.18, −1.25)	−3.30 (−7.52, 0.92)
**Employment status (Ref=employed)**						
Self-employed	0.58 (−3.63, 4.80)	−1.80 (−5.87, 2.27)	0.71 (−4.12, 5.54)	0.73 (−3.35, 4,81)	−2.11 (−5.71, 1.49)	0.35 (−3.35, 4.05)
Still at school	−1.78 (−5.86, 2.31)	−0.38 (−4.33, 3.57)	−3.42 (−8.11, 1.27)	−1.33 (−5.29, 2.63)	−1.16 (−4.65, 2.33)	−1.14 (−4.73, 2.44)
Volunteering or doing unpaid work	−5.95 (−12.97, 1.07)	−3.22 (−10.00, 3.56)	−2.80 (−10.85, 5.25)	−5.67 (−12.46, 1.13)	−3.18 (−9.18, 2.81)	−2.70 (−8.86, 3.47)
Unemployed: no structured activities	−10.41*** (−15.00, −5.82)	−9.95 (−14.38, −5.51)	−13.31*** (−18.58, −8.05)	−7.74*** (−12.18, −3.29)	−7.45*** (−11.37, −3.53)	−7.65*** (−11.69, −3.62)
Other, mainly commission based	0.04 (−10.10, 10.18)	−5.61 (−15.41, 4.19)	−20.93*** (−32.55, −9.31)	−2.01 (−11.83, 7.81)	−6.13 (−14.79, 2.53)	−14.37*** (−23.27, −5.47)

CI: confidence interval; U=unadjusted regression coefficients.

* *p*<0.05

** *p*<0.01

*** *p*<0.001.
